# The COVID-19 Pandemic Challenged Nurse Managers' Daily Leadership Work: A Qualitative Study

**DOI:** 10.1155/2023/8191426

**Published:** 2023-12-20

**Authors:** Annika Ahlqvist, Anu Nurmeksela, Tarja Kvist

**Affiliations:** Department of Nursing Science, Faculty of Health Sciences, University of Eastern Finland, Kuopio Campus, PL 1627, Kuopio 70211, Finland

## Abstract

**Aim:**

To explore and describe nurse managers' experiences of how the COVID-19 pandemic affected their leadership work.

**Background:**

As frontline leaders in healthcare, the work of nurse managers was dominated by the global COVID-19 pandemic in recent years.

**Method:**

Semistructured interviews were carried out during autumn 2021 at a Finnish university hospital. Twelve nurse managers participated in video call interviews, and the data were analysed by inductive content analysis.

**Results:**

Four main categories of COVID-19 effect on the nurse managers' leadership work were identified: increased work requirements; changes in work content; crisis communication; and human resources. The main categories included a total of 14 subcategories.

**Conclusion:**

The complexity and range of nurse managers' leadership work during the pandemic were emphasized. Nurse managers performed several important tasks that ensured ongoing delivery of healthcare services. Flexibility, ability to solve problems, capacity to tolerate uncertainty, pressure and rapidly evolving situations, excellent crisis communicational skills, and an ability to address the diverse needs of their staff were key competencies that nurse managers relied on during this period. *Implications for Nursing Management*. Nurse managers' experiences of COVID-19 can inform practice, policies, nursing, and nursing leadership education in relation to future crises.

## 1. Introduction

Societies and healthcare systems all over the world faced unprecedented challenges at the beginning of 2020, when COVID-19 was categorized as a global pandemic [1]. The unknown virus and its unpredictable progression affected different countries in various ways and required significant efforts from healthcare organizations, professionals [2], and governments [3]. Healthcare leaders had to respond quickly and embark on leadership approaches suited to a time of crisis.

In crisis management, five different phases have been recognized [4]: detection; probing and prevention; damage containment; recovery; and learning. These phases have been apparent during the COVID-19 crisis, and we have proceeded into a postpandemic learning phase, as COVID-19 is no longer defined as a global health emergency [5]. By recognizing phases and managing each of these stages appropriately, organizations can increase their crisis management capability [4]. In the nursing context, good crisis leadership is needed for clear, timely, and honest communication in sharing information. Furthermore, fair decisions and prioritization by leaders are required. Leaders are also expected to exhibit a high degree of collaboration, competency, and trust-building [6].

In this study, we view nurse managers' leadership work as whole, since leadership and management are closely interwoven with each other [7, 8]. In times of crisis, such as the COVID-19 pandemic, nurse managers play an essential role in both leading and managing [9]: setting directions, aligning teams, and motivating staff in terms of leadership as well as planning, organizing, controlling, and solving problems as a part of management [7]. Both parts shared the same aim [7]: influencing team members to work collectively in order to survive through the COVID-19 pandemic; in this study, we use the term leadership.

Nurse managers play a vital role in leading departments and healthcare teams on the frontline. They are responsible for organizing nursing, maintaining a good working environment with multi-professional collaboration, and promoting nurses' personal development to achieve good nursing outcomes [10]. Nurse managers also have an important duty implementing evidence-based practices, organizing units and daily work, offering support, and recruiting staff [10]. In particular, during the pandemic, decision making and strong leadership were highlighted in nurse managers' work. They were responsible for decisions directly connected to staff, patients, and the provision of nursing care [11]. Performing internal crisis communication [12, 13], being visible and decisive, leading with empathy, and easing nurses' stress were also key tasks for nurse managers providing leadership through the crisis [13].

Considering the challenge faced by the staff of fighting COVID-19 while maintaining safe and ongoing delivery of patient care, the importance of nursing leadership cannot be overstated. Nurse managers' visibility, understanding of the situation, care for the working environment, and relational leadership style were seen to support nurses' resilience during COVID-19 [14]. An effective nursing leadership style is also crucial for high-quality patient, nursing, and organizational outcomes [15, 16].

In previous studies, COVID-19 has been described from the nurse managers' perspective as challenging, stressful, and pressured [17]. Rapidly changing circumstances are challenging, but the pandemic's global nature and its implications, such as widespread lack of equipment, made the situation even more complex for those in leadership roles [9]. Working practices had to respond to uncertainty and continually evolving guidance. However, while novel problems without known solutions present challenges to management, they can also lead to proactive problem solving [18]. Carefully planned development and improvement projects had to be put aside, and new plans had to be created on the ground [19].

Confusion and lack of understanding of, and inability to control, the rapidly evolving situation challenged nurse managers at the beginning of pandemic, causing psychosocial symptoms such as anxiety. Nurse managers also reported fatigue, tiredness, and exhaustion [17]. Their work has been described as emotionally fluctuating, with feelings ranging from fearful and unable to concentrate to hyperaware, energized, and daring [19]. Despite the hardships, some nurse managers felt their skills in management, decision making, and communication developed during the pandemic [17], and they gained more insight into how to be better prepared in the future regarding both the workforce and other resources [11].

Good management of human resources is essential. Because of changes in staff and the varying demands arising from the care of new patient groups, staff management during the pandemic was complex [18]. COVID-19 differed from earlier crises because it included the recruitment of volunteer and non-volunteer staff, arranging flexible work schedules, and continually rearranging staff as the situation evolved [20]. Taking care of staff needs, both material and psychosocial, was one of the nurse managers' priorities [18, 20].

Communication is known to be an essential part of crisis management [6]. Challenges in communication arose during the pandemic when information was not available at the right time, was contradictory, or came from different sources. Nurse managers had to create new strategies to keep staff up to date. Ensuring that information was clear, concise, and truthful was an important task [18]. Communication was a particular challenge for leaders working remotely, as they were uncertain whether the essential information reached employees promptly. They also expressed concerns regarding employee's being able to contact them easily [21].

Research on nurse managers' experiences during the pandemic has been growing in the last couple of years. However, there is still a need for diverse data about nursing leadership during the period to create wider and more in-depth view of the phenomenon. This study offers an important perspective on nursing leadership in the context of specialized university hospital level healthcare. Even though the pandemic is no longer defined as a global emergency, there is a need to act and actively learn from past efforts [5]. Studying real-life experiences of frontline nurse managers and recognizing their importance in performing crisis leadership is required to develop future crisis preparedness on the global, national, and organizational scale. It is hoped that this study will contribute to a deeper understanding of the topic as it describes broadly the elements and demands of leadership during a time of crisis.

## 2. Methods

### 2.1. Aim

The aim of this study was to explore and describe nurse managers' experiences of daily leadership work during the COVID-19 pandemic. The research question was “How did nurse managers experience the effects of the COVID-19 pandemic in their daily leadership work?”

### 2.2. Study Design

A qualitative descriptive interview design was used.

### 2.3. Participants and Data Collection

All the nurse managers (*N* = 46) of university hospital studied received an invitation letter by e-mail from the hospital's chief nursing officer at the end of May 2021. The inclusion criterion was working as a nurse manager during the COVID-19 pandemic. A total of 12 nurse managers registered for the study. Nurse managers were chosen by purposeful sampling to ensure that they had the required experience of the study phenomenon [22].

The interview guide dealt with the nurse managers' experiences of the pandemic and especially its effects on nursing leadership work (Table 1). The interview guide was designed around the research question and based on previous studies (e.g., [11, 23]) related to the theme. The guide was pretested with one nurse manager to determine the understandability and functionality of the questions and revise them if needed [24]. As no changes were required, the pretest data were included in the analysis. The interviews were carried out by the first author using online video calls undertaken between August and September 2021, lasting 41 minutes on average (range: 31–61 minutes). 61 pages of transcript material were received. The timing of the interviews fell between the third and fourth waves of the pandemic [25], during what can be described as a momentary recovery phase [4] in the COVID-19 crisis.

### 2.4. Study Setting

Data were collected from the nurse managers in one of five public university hospitals in Finland using a semistructured interview. The university hospital offers specialized medical care for approximately 250,000 people and approximately 3,000 nursing professionals work there. The number of confirmed COVID-19 positive patients in the hospital ranged daily from 0 to 50 from the 28th of March, 2020, to the 1st of September, 2021 [25]. The hospital in the study was not the one taking care of the highest number of COVID-19 patients in Finland.

The nurse managers were first-line managers and worked in different specialties at the university hospital, including acute care, inpatient wards, and outpatient departments. Five wards took care of suspected or positive COVID-19 patients daily, weekly, monthly, or intermittently. Other wards did not participate in direct care, but the pandemic still affected them in multiple ways as new instructions had to be implemented, preparations and changes related to COVID-19 had to be executed, the staff's need for support increased, and overall uncertainty prevailed.

### 2.5. Data Analysis

The first author transcribed the recordings into a written form verbatim without using any transcription programs. Interviews were listened to several times to ensure the validity of the data [22]. The data were analysed manually using inductive content analysis, as it is well suited to examining new and interesting phenomenon [26, 27]. The written material was carefully read through several times as descriptive meaning units such as phrases and paragraphs were chosen [27]. Care was taken that the selected meaning units were large enough to form a clear picture of the interviewees' thoughts and context. Latent content was not analysed. After collecting meaning units into a file, they were condensed and anonymized. The condensed data were organized into appropriate subcategories based on their similarities, differences, and cohesiveness [22, 26, 27]. The abstraction process started and formed subcategories were divided into appropriate main categories based on their content [22, 26]. The collected data were found to be saturated when themes became repetitive and new information no longer emerged [22].

The first author conducted the interviews and undertook the preliminary data analysis. All authors were involved in evaluating the primary meaning units, condensing data, and the categorization process. The results were discussed until a consensus was reached. Early stage and experienced researchers in nursing science carried out all of the phases of the research. Example of the analysis process is shown in Table 2.

### 2.6. Rigour

Throughout the process, five essential principles of research trustworthiness were followed: credibility, confirmability, dependability, transferability ([28], by [22]), and reflexivity [22, 29]. To ensure credibility, all the key aspects of the study, such as the nurse managers' demographics, research environment, and analysis process have been described carefully and truthfully [22]. All the nurse managers in the study met the defined inclusion criterion of having the required experiences of the study phenomenon (leadership during the COVID-19 pandemic). Video and audio recorded interviews and verbatim transcription provided a solid basis for the analysis process, which was meticulously carried out.

To ensure confirmability and dependability of the study, all the members of the research team systematically and actively engaged in all stages of the research process from planning to writing, to allow differing interpretations of the data [29]. Data analysis and results were thoroughly evaluated and subjected to the research teams' critical discussion before consensus on the best possible interpretation was achieved [22]. Nurse managers were not asked to offer feedback on the study afterwards [29]. An example of the analysis (Table 2) and detailed description of the process are presented to demonstrate the stages. Results were descriptively described, and to corroborate the authenticity of the interviewees' personal views, direct quotations were utilized extensively [22, 29]. Quotes were chosen based on their illustrative value.

To strengthen transferability, we present detailed information about the research setting and the study participants [22, 29]. However, due to confidentiality, details about nurse managers' wards were not given, and this may weaken the transferability of the study. It is also important to note that this study was conducted in between the third and fourth waves of the pandemic, in autumn 2021, and the interviews reflect the nurse managers' experiences during this exceptional period.

Regarding reflexivity, the first author, who conducted the interviews, had experience of nursing during the COVID-19 pandemic, but within a different organization and in a different professional position than the interviewees. The coauthors have worked as nurse managers and are experienced researchers of nursing leadership. This provided a valuable perspective that contributed to a deeper preunderstanding of the study phenomenon. Personal experiences, thoughts, and professional identity were recognized and considered through the research process to meet the criteria of reflexivity [22, 29].

### 2.7. Ethical Considerations

The study design was reviewed and approved by the Committee of Research Ethics of the University of Eastern Finland (Decision Number: 2/2021). Permission for the research was granted by the university hospital in accordance with its permission guidelines. The study complied with the principles of the Finnish National Board on Research Integrity [30] and the EU General Data Protection Regulation [31]. The interviewees received an information sheet in which they were provided with details of the study, taping interviews, and voluntary participation. Each interviewee gave their informed verbal consent. To ensure confidentiality and a safe interview environment and prevent identification of the nurse managers, their wards or units were not specified in the study.

## 3. Results

### 3.1. Demographics of the Nurse Managers

A total of 12 nurse managers participated in the study. Their average age was 51 (range: 38–61). Nine of the participants were female, and three were male. Seven nurse managers had a master's degree, and five of them had a bachelor's degree plus additional training. The average length of work experience as a manager was 9 years, ranging from 3 months to 26 years.

### 3.2. COVID-19 Pandemic Effects on Nurse Managers' Leadership Work

**Figure 1:**
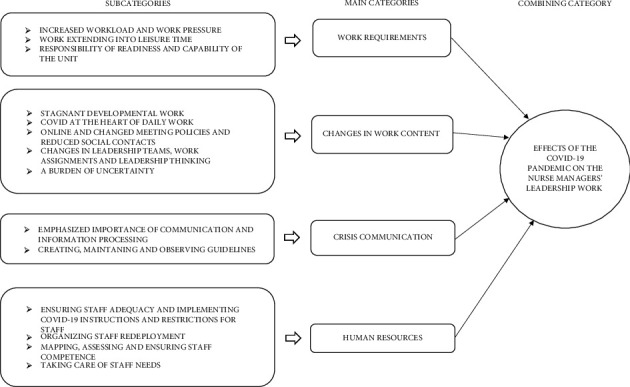
Effects of the COVID-19 pandemic on nurse managers' leadership work.

The content analysis produced one combining category, four main categories, and 14 subcategories, as shown in Figure 1.

The effects on the nurse managers' leadership work fell into four main categories: increased work requirements; changes in work content; crisis communication; and human resources.

#### 3.2.1. Increased Work Requirements

Nurse managers reported *increased workload and work pressure*. Their work had clearly become more stressful and pressured. Some stated that the pandemic represented a difficult stage in their working life and there is a real need to find the joy in work again, from new and pleasant things. Others felt inadequate regarding growing patient queues and throughput as well as the inability to respond to the need for treatment. A strong feeling of responsibility arose from the need to ensure adequate daily nursing staffing levels. Unfinished work caused some to constantly feel guilty. Additional work pressure also arose from other parties, such as colleagues and higher management. Different wards had different challenges, protocols, and uncertainties related to organizing nursing staff and COVID-19 patients. Pressured circumstances, the need for staff, and an inability to understand other wards' way of acting sometimes caused confusion and tension between some nurse managers and increased stress levels. The nurse managers also felt that demands from higher management for ongoing organization-level developmental work in their wards was unnecessary.

*“We had other situations (acute matters related COVID-19 and the ward's basic functioning) that needed prioritizing, and at the same time we were asked if we had completed all the tasks related to our other projects. Personally, I told them it was unreasonable.”* NM10

A few of the nurse managers mentioned *work extending into their leisure time*, and that it was sometimes hard to leave work behind. Phones had to be carried everywhere, even out of working hours in case someone called from work. In some cases, the nurse managers had to go into work on a day off or call into work to ensure that all the protocols were clear and to avoid any safety risks for staff and patients.

To ensure the *readiness and capability of the unit*, nurse managers had to set up crisis plans, ensure adequate expertise was available for various new functions, plan patient placements and ward capacity, prepare to receive COVID-19 patients, and purchase different equipment in advance (e.g., protective equipment and laptops, cameras, and headphones for staff who were working remotely). Some wards even needed construction work and the redesigning of facilities or had to be relocated to function properly and meet the new hygiene and isolation requirements. Not all facilities were fully suitable, which caused daily organizational challenges as patients with infection symptoms had to be taken care of in the same spaces as those in more fragile situations. Some nurse managers faced significant changes as they had to shut down their unit partly or completely and then restart the unit's operations again. Decisions were made quickly and had to be implemented within one or two days. A lot of organization and fast action was needed in order to make everything work within a such short time frame.

#### 3.2.2. Changes in Work Content

Several nurse managers reported *stagnant developmental work*. There was not enough time to promote normal developmental work related to nursing, ward activities, or priorities set by the organization. Daily priorities were mainly concentrated on surviving COVID-19-related matters, and previously defined goals and other tasks had to be put on hold.

*“One thing that has received less attention is developmental work. —… There hasn't been time to stop and think for a longer period about how we could develop something, or even time to come together in a bigger group to discuss things.”* NM4

*COVID-19 was at the heart of most nurse managers' daily work*, and COVID-19-related practical matters took up most of their time. The workload was described as constant organizing, guiding, and planning nursing requirements, coping with daily situations, arranging and rearranging, answering questions, being on the phone, “fire-fighting,” surviving daily tasks, taking care of staff resources, and ensuring operational maintenance. It was full-time “COVID thinking” with little opportunity to think about anything else. However, some nurse managers perceived the basic leadership work stayed the same, as it was the same kind of organizing and taking care of staff as usual. In specific units, there were fewer concerns compared to those in somatic care, as their patients were generally basically healthy. Previous experience of crisis situations allowed a sense of calm and prevented anxiety in the context of an ever-changing situation. Staying composed was emphasized and working partners with prior leadership experience were a valuable asset. Some nurse managers had to make big decisions about ward procedures (e.g., planning COVID-19 patient flow in the hospital and starting new processes like taking COVID-19 samples) and would have appreciated collective organizational support for addressing these issues.

*“Sometimes it would be good to stop and think a bit about how things are going in different situations before we start to plan the next one. We do not actually even plan, there is no time for planning, we just jump to the next task. Nowadays planning is quite minimal and then it is implemented straight away.”* NM10

Tightened restrictions to avoid unnecessary contacts led to *online and changed meeting policies and reduced social contacts*. Non-COVID-19-related meetings and training were cancelled or went online. In one unit, the absence of weekly staff meetings caused the spread of unfounded rumours and raised unnecessary fears. Some wards increased the number of meetings and started sending out daily communications to ensure everyone was up to date. Some nurse managers' line meetings changed from monthly to weekly, which increased and advanced cooperation with multi-professional partners. Online meetings were described as timesaving and helped the planning of daily work. Some managers commented that the new working environment suited them better. On the other hand, some felt that decreased face-to-face meetings hampered network functionality and the availability of peer support and made them feel isolated.

*“This Microsoft Teams -world is what it is. There are good things in it, and it might be this will stay as a part of our working life so that we don't spend so much time travelling places anymore. But I would personally need a degree of peer support, sense of community and things like that.”* NM2

COVID-19 also resulted in a demand for *changes in leadership teams, work assignments*, *and leadership thinking*. A few assistant nurse managers were moved into different units or onto different tasks. Some nurse managers were allocated new temporary working partners to replace the assistant nurse managers, while others had to take on assistant nurse manager duties as well as carrying out their own leadership role. A few nurse managers also carried out nursing duties alongside their own work. Others were prepared to take on responsibility for other units if needed. Some nurse managers described how the pandemic resulted in changes and growth in their leadership thinking, as anticipating new challenges and crisis awareness became more central to their work. The pandemic also changed working practices in nursing, leading to new ways of working. Remote working has the potential to become a regular part of working practice in some places and a tool for supporting staff wellbeing. This now needs continual revaluation and reliable and easy to use digital platforms and devices to become part of future operational practice.

*“I am here as a nurse manager, as a ‘producer of artwork.' So, my job is to ensure that ‘artists' have everything they need to work as smoothly as possible. It has required a rethinking of things and implementation from different perspectives.”* NM12

*A burden of uncertainty* arose from the incomprehension, unpredictability, and continual uncertainty around the COVID-19 pandemic. There was an expectation that the nurse managers were always up to date with guidelines and instructions, even though they did not always have the answers. The prolonged nature of the pandemic and a fear of going through the same misery again took the joy out of some nurse managers' work.

#### 3.2.3. Crisis Communication

Nurse managers had to adapt to *processing and sharing new information and*, *in their narratives, the importance of communication* was *emphasized*. The volume of new information they received was described as enormous and required a lot of processing, internalizing, and studying. Some instructions were contradictory, and it took time and energy to determine the most important information, together with how to proceed. The nurse managers highlighted how they had to constantly keep staff informed, as they felt that this was a priority. The staff's need for information was described as endless. Because of the daily changing nature of the information required, some nurse managers started to send out daily communications or have an information briefing at the beginning of every shift. However, the nurse managers felt that problems occurred especially at the beginning of the pandemic and organization-level communication improved over time.

*“The amount of new information and guidelines was insane, and it was our job to understand and implement it.”* NM7

*“If we think about this local newspaper, information there was updated, but the information channel that I followed and was instructed to follow was not always up to date. There were two kinds of information. And that caused extra confusion.”* NM10

Nurse managers were responsible for *creating, maintaining, and observing guidelines*. They made sure that COVID-19-related practices such as hand hygiene, safe distancing, and meeting restrictions were implemented and followed. However, organizational hierarchy and discrepancies in the information from different doctors sometimes made it difficult to put final instructions (e.g., about hygiene policy and how to work with COVID-19 patients) into practice.

“*It took a long time to think and refine the COVID-19 related procedures so that we found a clear and correct course of action (in the ward).*” NM12

#### 3.2.4. Human Resources

*Ensuring staff adequacy and implementing COVID-19 instructions and restrictions for staff* was demanding, time consuming, and added more pressure to the nurse managers' workload. Increased staff sick leave due to COVID tests and reactions to vaccinations affected daily planning, and it was not always easy to find deputies. Nurse managers had to consider when additional staff were required and sometimes worked other shifts as well as their own. Many questions arose from the staff regarding COVID-19 infectivity, testing, quarantine policy and symptom assessment, salary, and income arrangements, sick leave policy, the effect of family members' infections, and being exposed to the virus. Some nurse managers offered daily guidance, but at times unclear instructions made this difficult. Decision making required careful consideration because solutions were not always known. One nurse manager felt new, clearer testing and sick leave instructions shifted the responsibility away from the nurse leaders and significantly helped the daily workload.

*“At this moment a big part of the working day is spent trying to organize staff for each shift, taking into account COVID preparedness. —… In the summertime staff resources are very minimal and we have been hanging on a thread: will we get COVID patients or not?”* NM3

*“I think in our hospital we have had quite unclear instructions about determining the level of exposure. I had to ask about those separately, because many people read them differently and there was too much room for interpretation.”* NM2

Changes in ward operations meant *organizing staff redeployment*. Nurse managers had to find out where to relocate staff, what would be similar work, and where they would be needed, while also considering the needs of at-risk groups. Leading staff members in the COVID-19 era posed different problems compared with before the pandemic because they could be placed anywhere in the hospital, and they could be part of a new process. Unclear and changing timelines for getting staff back were also a challenge.

*“70% of my staff were relocated and I was the one who had to organize those places for them —… It (information about getting staff back) was unclear and vague. First it was two weeks and finally it turned out to be seven weeks, and then many other things came up.”* NM10

*Mapping, assessing, and ensuring staff competence* were some of the nurse managers' duties. In some wards, some staff had to be transferred to another unit and familiarize themselves with reception, the ward, COVID-19 cohort, or intensive care work. Under time pressure, nurse managers had to assess an employee's educational background, competence, strengths, willingness, and personal risk factors and then make a decision at short notice. They also needed to coordinate the roles of new staff, taking the responsibilities required into account without overloading individuals. Nurse managers also had the responsibility of ensuring that the staff attended COVID-19 training sessions. They needed to make sure that every staff member received the fast-track training provided by the organization. This required a lot planning and arranging. Some nurse managers said that the training offered was not tailored to their unit's specific needs. The staff were proactive in their participation, but training amid uncertainty was demanding.

The nurse managers said that *taking care of staff needs* was essential but hard work. It included listening, calming, addressing insecurities, being present, receiving feedback and critique, having one-to-one conversations, actively maintaining staff wellbeing, organizing workplace picnics, limiting extra work tasks, and organizing joint meetings with the occupational health department, staff, and nurse executives. Some described how the chaotic nature of the COVID-19 situation especially at the beginning detracted from supporting staff. In addition to leading with knowledge, a holistic approach was needed because so much was also happening in the staff's personal lives. One nurse manager said that hospital maintenance staff and secretaries turned to her for support because their leaders were working remotely and were not readily available. This caused extra stress but also highlighted the importance of being present in-person.

*“I think we (nurse managers) need more tools to support nurses' work-wellbeing. I feel that during my studies we did not have enough discussions about these sorts of things. In the healthcare sector we need concrete approaches and education for leaders, how to help and support. And of course, for the staff, how to take care of their own resources”* NM1

## 4. Discussion

This study aimed to explore nurse managers' experiences during the COVID-19 pandemic to obtain a deeper understanding of what happened. The research hospital was one of five Finnish university hospitals that offer high-quality specialized medical care, execute international research, and have an important responsibility for training and educating upcoming healthcare professionals [32]. They have a critical position in Finnish healthcare infrastructure, offering advanced level care, as emphasized during the COVID-19 pandemic. Examining phenomena through this perspective gives an opportunity to view a context in which many crucial medical and nursing functions are located. As the results show, the COVID-19 pandemic affected nurse managers' leadership work in various ways. Delivering crisis communication, adapting to changes in work content and increased requirements, and taking care of human resources were highlighted in their stories.

According to the interviews, crisis communication was pivotal during the pandemic. Collecting, internalizing, and synthesizing information and keeping staff well informed was a continuous and time consuming, but essential, task. Sometimes confusion and the changing nature of the information available made the situation difficult for nurse managers as described in other studies [18, 34]. In this study, it was reported that organizational communication improved over time, but at the beginning of the pandemic information differed between newspapers and internal channels, which caused confusion among professionals. Thus, external information sources being more up to date than organizational sources may weaken employees' trust in the information received from the organization. In advance of a future crisis, it is important that organizations develop clear information-sharing forums and protocols. Nurse managers are in a central position in internal crisis communication [12] and, in order to avoid ambiguity and ease nurse managers' workloads, the quality and coherence of information they receive is extremely important. Internal crisis communication also requires more dialogue and emotionally supportive elements [12], as leaders' communication has great importance for supporting nurses' resilience in a crisis [14]. Evaluating and enhancing communication skills, both in-person and on digital platforms, is essential for nurse managers in the future. This is supported by the knowledge that in some fields, the pandemic required leadership and communication solely through digital systems [34], as also reported in this study.

COVID-19 challenged and changed nurse managers' daily leadership work and its content in multiple ways, as uncertainty, rapidly changing situations, and new problems often presented themselves. Time has been described as an unpredictable spiral [35]. Even though the organization of nursing, units, and staff is part of every nurse managers' work [10], the ability to make decisions and implement them at short notice was in a crucial role [11] as COVID-19-related matters were at the heart of the work. Navigating through insecurities and the changing environment, leading nurses, and carrying the responsibility of successful ward operations was described as a multidimensional task in our nurse managers' stories. There were also sudden changes in leadership teams which, at times, meant greater responsibility and more tasks placed on nurse managers' shoulders, even requiring them to undertake nursing duties. In the future, it would be beneficial for organizations to evaluate thoroughly the allocation of limited resources and division of tasks, allowing the nurse managers to focus on the essential aspects of leadership work. Along with the challenges, the pandemic was also said to have developed their crisis managements skills, as reported in earlier studies [11, 17]. Nurse managers in the study stated that circumstances demanding isolation or distancing made them think about practices from a different perspective. It led to changing procedures and enabling nurses to work remotely at times, which was not possible previously. This rapid digital leap meant more flexible services for patients in certain areas. New visions have the potential to highlight new approaches for the future and encourage the development of more flexible working practices—for nurse managers, nurses, and patients.

During the pandemic, nurse managers' work requirements and workload rose, work extended into leisure time, and feelings of responsibility and guilt were present from time to time. Ensuring patient safety and staff safety and the ability to keep ward functions running were critical tasks and enhanced in the study. At times, nurse managers' work was described as pressured and stressful, as documented in earlier studies [11, 17, 34]. Nurse managers, as well as their nurses, needed great resilience, the ability to reflect, cope, commit, solve problems, develop coping strategies and self-awareness, and trust in the future [14] to overcome the pandemic. It is important to consider that leadership during the COVID-19 pandemic was different than that during other crises, e.g., natural disasters, due to its unpredictable nature and long-lasting duration [36].

Leading human resources meant occasionally making hard decisions while assessing staff adequacy, competence, and redeployment. Nurse managers had to adapt to the fact that their staff were relocated in the hospital and leadership was sometimes performed remotely. Taking care of the needs of staff is part of nurse managers' daily leadership work [10], but during the crisis, the importance of this was particularly emphasized [13, 18, 20, 34] as needs were more acute and providing support also meant broader scale human resources leadership. Along with work, challenges and worries in their private lives also affected employees and nurse managers had to consider situations holistically. Relational leadership styles, such as authentic leadership [36], have been recognized as effective during the pandemic [14]. In this study, other professionals relied on nurse managers because their own leaders were not always readily available: being present was emphasized.

In crisis processes, it is important to enter a learning phase and to be prepared for future crises at all levels of the organization by providing opportunities for open and safe collective reflection on past experiences [4]. Organizations could benefit from studying which practices were beneficial and should be continued or developed and what tasks could be taken care of collectively, centralized, or even paused during a time of crisis. Giving nurse managers sufficient resources and enough support would be beneficial [17], especially in the acute stage of a crisis. Creating pathways for regularly sharing updated information with colleagues at all levels could reduce stress and promote nurse managers' collective wellbeing and feeling of partnership. Overall, analysing and learning from what happened is a fundamental part of healthcare systems' future resilience but unfortunately is easily forgotten when situations return to normality [2].

Exploring the nurse managers' experiences during the COVID-19 pandemic helps us understand the demands made on their time and makes their efforts more visible. A better understanding will help organizations develop functional working practices and policies and appreciate nurse managers by verifying their experiences. It is important to ensure that they want to continue in their professional leadership positions [34]. Further research by gathering more specific information from nurse managers about what actions were successful, what failed, and what needs further development would be informative for organizations. This study highlights nurse managers' experiences of various demands they faced during the pandemic period. Along with leadership work and other work-related themes, strengthening knowledge of personal and work wellbeing and coping strategies for nurse managers is valuable. As some nurse managers stated, the sense of community and joy from work decreased during the pandemic. Conducting in-depth interviews could give us deeper insights.

The unique experience of COVID-19 presents an opportunity to develop the study of phenomena and crisis preparedness for the future. Well-organized and managed health systems with appropriate resources have more resilience in crisis situations [2] and a sense of threat can be transformed into healthcare professionals' empowerment [37]. In this study, nurse managers reported gaining insights and learning experiences from the period. In well-prepared crisis or disaster plans, it is crucial that those in leading positions have sufficient skills to manage these situations [2, 38]. Reedy et al. [39] have shown that not all nurse managers have adequate and consistent education for crisis and disaster management. Exploring and assessing nurse managers' crisis management needs and skills more widely in qualitative and quantitative ways, creating educational intervention and revaluation, could be beneficial at organizational and national levels when preparing for future crises.

### 4.1. Strengths and Limitations

There are both strengths and limitations to this study. One strength is that the nurse managers were open and motivated to share their experiences, which led to rich and abundant data. The nurse managers represented widely different specialties within the hospital, enabling the study phenomena to be viewed from various perspectives. The timing of the interviews meant the nurse managers had the opportunity to consider the possible course of the pandemic.

Regarding limitations, the first author conducted a preliminary analysis of the data, although all authors were involved in evaluating primary meaning units, condensing data, and the categorization process. The study was conducted within a single specialized healthcare organization; therefore, the results may not be transferable to a different healthcare setting, region, or country. The variety of wards placed different demands on nurse managers. The results present a wider reference group, and the experiences of different specialties are not classified separately. Although not all the nurse managers were directly responsible for COVID-19 patients, the pandemic still affected their work in multiple ways, especially at its onset.

## 5. Conclusions

This study provides a comprehensive and detailed description of nurse managers' daily leadership work and its demands during the COVID-19 pandemic. A university hospital during a pandemic was a remarkably complex operational environment for leadership work. Nurse managers performed several important tasks that ensured ongoing delivery of healthcare services. Flexibility, ability to solve problems, capacity to tolerate uncertainty, pressure and rapidly evolving situations, excellent crisis communicational skills, and an ability to address the diverse needs of their staff were key competencies that nurse managers relied on during this period.

In relation to preparing for possible future crises, it is important to allow nurse managers to concentrate on the core aspects of their leadership work. This could be done by allowing the managers to suspend routine development work and to delegate some of their tasks, especially at the beginning of a crisis, according to a predetermined plan. Recognizing the nurse managers' valuable work and supporting them during demanding times is important for a sustainable and flourishing working life.

## 6. Implications for Nursing Management

The global pandemic has offered nurse managers a unique and historic experience of frontline crisis management and leadership. It is important to systematically reflect on these experiences that are valuable in developing healthcare policies, organizational practices, and nursing as well as nursing leadership education. Clear and well-organized crisis plans, including a clear division of work and ward procedures, could support nurse managers' leadership work in future crises. Informative and clear crisis communication and staff support are vital. In addition to occupational health care, organizations could benefit from establishing centralized and easily available low-threshold crisis support systems, such as opportunities for psychosocial support for the healthcare professionals.

## Figures and Tables

**Table 1:** Interview guide.

Main question
(i) Please, describe how the COVID-19 pandemic has affected your leadership work?
Examples of probing questions
(i) How has the COVID-19 pandemic changed your daily leadership work?
(ii) Can you tell, what is new about your leadership work during the pandemic?
(iii) What things in your leadership work haven't you had time to do or have had less time to do during the pandemic?
(iv) What do you think are the things that have been highlighted in your leadership work during the pandemic?
Final question
(i) What do you think and how do you feel, is there anything else that you would like to discuss, add, or tell me more about?

**Table 2:** Example of the analysis process.

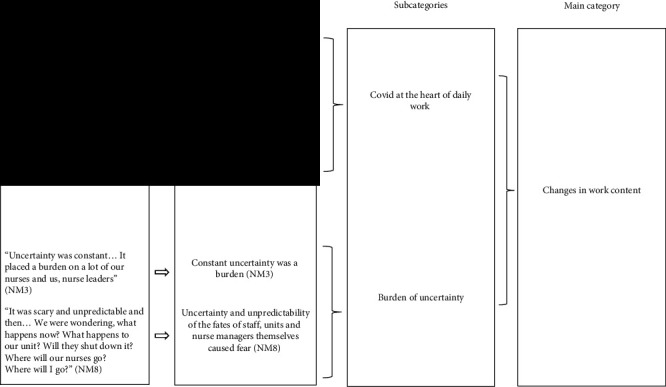

## Data Availability

Research data are not shared due to data's sensitive nature.
